# Sulphuric-Acid Paste in the Treatment of Epithelioma of the Face*Read before the Medical Society of the District of Columbia, April 18, 1894.

**Published:** 1894-09

**Authors:** E. Oliver Belt

**Affiliations:** Washington, D. C., Lecturer on Ophthalmology and Otology, Howard University, and Ophthalmic and Aural Surgeon to Freedman’s Hospital


### SULPHURIC-ACID PASTE IN THE TREATMENT OF EPITHELIOMA OF THE FACE.*

By E. OLIVER BELT, M.D., Washington, D. C.,

Lecturer on Ophthalmology and Otology, Howard University, and Ophthalmic and Aural Surgeon to Freedman's Hospital.

Epithelial carcinomata about the eyelids, brow, nose, and cheek are not uncommon. The clinical history of an ordinary case is about as follows: Beginning usually after middle life as a small, flat, wartlike growth, the new formation appears very innocent at first, attracting but little attention, perhaps, until some years elapse, when it begins to ulcerate. The ulceration may heal temporarily, only to begin anew later on, when it suddenly begins to spread, attacking all kinds of tissue, sometimes destroying the eyeballs, nose, and bones of the face, the patient being in a most pitiable condition until relieved by death. Fortunately most of these cases are found in very old people, who die of natural causes before the growths give much annoyance. Occasionally, however, we see them in patients between forty aud sixty years of age, with whom they may become a very serious trouble, and even destroy life years before the allotted time. By proper treatment before too much tissue is involved, epithelioma of the face can unquestionably be eradicated, and yet we see now and then cases that have passed beyond all help of the physician or surgeon. Inquiry into the history of these hopeless cases will usually elicit the fact that the patient has allowed the trouble to reach this stage owing *to the dread of having it cut out with a knife*, which may have been recommended by his physician, or to escape this he has applied some "cancer cure" that he has heard of or seen advertised, which has served only to irritate, and often rapidly extend the disease. Most authorities recommend excision, and no one doubts its efficacy; but if the dread of the knife causes one to postpone treatment, it is well to try something unobjectionable to the patient and especially reliable; so we naturally turn our attention to local applications. We find many preparations recommended; among them may be named caustic

* Read before the Medical Society of the District of Columbia, April 18, 1894.

potash, citrine ointment, Vienna patse, and past of chlorid of zinc, arsenic, and sulphuric acid, as well as cautery. Of these some are very painful and harmful. Though the chlorid of zinc paste and the cautery seem to be the most frequently advised, my own experience leads me to call special attention to the value of a paste made of sulphuric acid and charcoal from saffron, in about equal quantities by weight. It is by no means a new remedy, having been used years ago by Velpeau, Michel, and others; but little has been written about it, and I do not think it is used now so generally as its efficacy justifies. I first saw it used by Dr. J. J. Chisolm, while I was his assistant at the Presbyterian Eye and Ear Hospital, where it was frequently employed during the three years that I was connected with that institution, and in no case did I see an unfavorable result.

In a recent letter from Dr. Chisolm he speaks of its use as follows: "I have been using carbo-sulphuric paste for thirty-five years in epithelial troubles of the face. It is one of the very old-time applications. I was taught its value as a student. The very fact that I continue to use it is because I know nothing quite so good. If properly made with flowers of saffron, which makes the finest of carbon, it becomes a tenacious paste that will stick to a raw surface and become a part of the slough or dry scab, when slough and paste come off together, leaving a newly healed surface, which delights the heart of the surgeon. It is a first-rate application, which every one should know about.

If the application is limited to the diseased tissue, it is not very painful unless the new growth is extensive. A few applications usually suffice, when a dry scab is formed, which peels off somewhat like a vaccination crust, leaving only a slight scar.

A single case illustrates its value as compared with some other methods. About eighteen months ago Dr. J. came to me to have a small epithelial carcinoma removed from the side of his nose near the inner canthus of the right eye. He told me that he had recently returned from New York, where he had taken his wife to a special hospital to have a similar trouble removed from her face. She was kept in the institution at heavy expense for three weeks, during which time caustics and poultices were applied. She was cured, but with a great deal of discomfort, loss of time, and heavy expense. Not being pleased with the treatment, the doctor, whose own case was pronounced worse, came to me to have the sulphuric-acid paste applied. Two applications were made in three days, and about the tenth day the growth came away entire, the doctor not having been kept from his practice more than a half-hour. I never advise this paste when the mucous membrane is involved. In all other cases that have not progressed too far, I like it as well as the knife, and think if physicians would use it with patients who postpone treatment from the dread of cutting, there would be few cases that reach the hopeless stage.—*The Medical News.*

